# Managing sleep disorders in ADHD: identification, consequences and clinical management

**DOI:** 10.1192/j.eurpsy.2023.70

**Published:** 2023-07-19

**Authors:** D. Wynchank

**Affiliations:** ISP ADHD, PsyQ, The Hague, Netherlands

## Abstract

**Abstract:**

Adequate sleep quality and quantity are essential for optimal occupational and psychological health as well as cognitive function. In up to 78% of adults with ADHD, several sleep disorders are associated.^1,2^ These include delayed circadian rhythm, insomnia, sleep-related movement and breathing disorders and altered sleep duration.^3,4^ Such sleep problems have consequences in the family and somatic health spheres. In the workplace, adult ADHD coupled with untreated sleep disorder leads to significant occupational impairment. Low employment status, unpredictable behaviour, relationship difficulties, mood lability, risk of injury and accidents are all described as potential associations, with far-reaching consequences. When ADHD is coupled with sleep disorders, cognitive performance deteriorates further and sickness absence is more common. The clinical presentation of the sleep disorders commonly associated with ADHD will be described in detail. State-of-the-art therapeutic interventions will be dicussed based on clinical experience and research findings from our Expertise Centre.

Wajszilber D, Santiseban JA, Gruber R. (2018). Sleep disorders in patients with ADHD: impact and management challenges. Nat Sci Sleep,14;10:453-480.

Van Veen MM, Kooij JJ, Boonstra AM et al. (2010). Delayed circadian rhythm in adults with attention-deficit/hyperactivity disorder and chronic sleep-onset insomnia. Biol Psychiatry. 1;67(11):1091-6.

Wynchank D, ten Have M, Bijlenga D et al. (2018). The association between insomnia and sleep duration in adults with attention-deficit hyperactivity disorder: results from a general population study. J Clin Sleep Med, 14(3):349–357.

Fayyad J, Sampson NA, Hwang I et al. (2017). The descriptive epidemiology of DSMIV Adult ADHD in the World Health Organization World Mental Health Surveys. Atten Defic Hyperact Disord, 91:47-65.

**Disclosure of Interest:**

None Declared

